# Medicines—Their Uses and Mode of Administration; Including a Complete Conspectus of the Three British Pharmacopocias, an Account of All the New Remedies, and an Appendix of Formulæ

**Published:** 1849-12

**Authors:** 


*Medicines—their Uses and Mode of Administration; including a complete conspectus of the three British Pharmacopocias, an account of all the new remedies, and an appendix of Formulæ.* By J. Moore Neligan, M. D., Edinburgh, M. R. J. A., etc., etc., etc. From the second Dublin edition. With additions, by Benjamin W. McCready, M. D., Prof. of Materia Medica and Pharmacy in the College of Pharmacy of New York, etc., etc., etc. New York; W. E. Dean, Printer and Publisher, 2 Ann St. 1849. 12mo., pp. 474.

The above transcript of the title page sufficiently describes the character and scope of the work. The American editor has added the "pharmaceutical preparations of the United States' Pharmacopocia, and short notices of such articles of our indigenous Materia Medica, as from their therapeutical value, or general employment, seemed to demand them." The work is recommended by Professors John B. Beck, and Theodric Romeyn Beck, and recommendations for a work of this kind could hardly come from higher and more reliable sources.

